# A Large Lung Abscess in an Electronic Cigarette User: To Drain or Not to Drain

**DOI:** 10.7759/cureus.37690

**Published:** 2023-04-17

**Authors:** Natasha Dudiki, Venu M Ganipisetti, Sashank Kolli, Simant S Thapa

**Affiliations:** 1 Pulmonary and Critical Care Medicine, Indiana University Health Ball Memorial Hospital, Muncie, USA; 2 Hospital Medicine, Presbyterian Hospital, Albuquerque, USA

**Keywords:** necrotizing pneumonia, e-cigarette and vaping product use associated lung injury (evali), percutaneous drainage of chest and abdominal colllections, electronic cigarette associated lung injury, • lung abscess

## Abstract

A lung abscess is a walled necrotizing infection involving the lung parenchyma, characterized by a cavitary lesion filled with fluid. It is usually caused by microbial infection with aspiration of oropharyngeal contents being the most common mechanism for primary lung abscesses. Secondary lung abscesses occur in the presence of predisposing lung conditions like bronchial obstruction, vascular or septic emboli or impaired host defenses. Lung abscesses caused by electronic cigarette use have gained relevance in the recent years since the outbreak of EVALI, that is, e-cigarette or vaping product use-associated lung injury, in 2019. First-line therapy involves prompt initiation of antibiotics given their success rate in the treatment of lung abscess in the current potent antibiotic era. Percutaneous aspiration and catheter drainage is considered a second line approach due to concerns for potential complications including catheter blockage necessitating repeat procedures, pneumothorax, hemothorax, hemoptysis, need for surgical intervention, infection of pleural space and bronchopleural fistula. We describe a case of a 21-year-old female with a history of electronic cigarette use presenting with a large left upper lobe lung abscess (14.5 x 8.5 x 13.3 cm) treated successfully with broad-spectrum antibiotics alone resulting in clinical and radiologic improvement.

## Introduction

A lung abscess refers to a focal cavitary area in the parenchyma filled with purulent or necrotic fluid in the parenchyma usually as a result of a microbial infection [1-2]. It is usually identified by chest imaging and is caused by liquefactive necrosis. Lung abscesses are categorized as primary or secondary [2]. A primary lung abscess is characterized by direct infection of the lung parenchyma in the absence of predisposing lung issues. This is mostly caused by aspiration or as a result of progression of areas of pneumonia, often caused by organisms like *Staphylococcus aureus*, *Streptococcus pneumoniae* and anaerobes [3]. Secondary lung abscesses are the result of predisposing lung conditions like bronchial obstruction, vascular or septic emboli or impaired host defenses. More recently, there have been a few case reports of lung abscesses associated with electronic cigarette use. First line of management involves appropriate antibiotics usually with good anaerobic coverage. Interventions like bronchoscopy to rule out obstructive etiologies, endoscopic drainage via bronchoscopy, percutaneous transthoracic drainage or surgical resection are reserved as second therapies in non-responding cases due to the potential complications associated with them. Here we describe a case of a 21-year-old female with a history of electronic cigarette use presenting with a large left upper lobe lung abscess (14.5 x 8.5 x 13.3 cm) treated successfully with broad-spectrum antibiotics alone resulting in clinical and radiologic improvement.

## Case presentation

**Figure 1:**
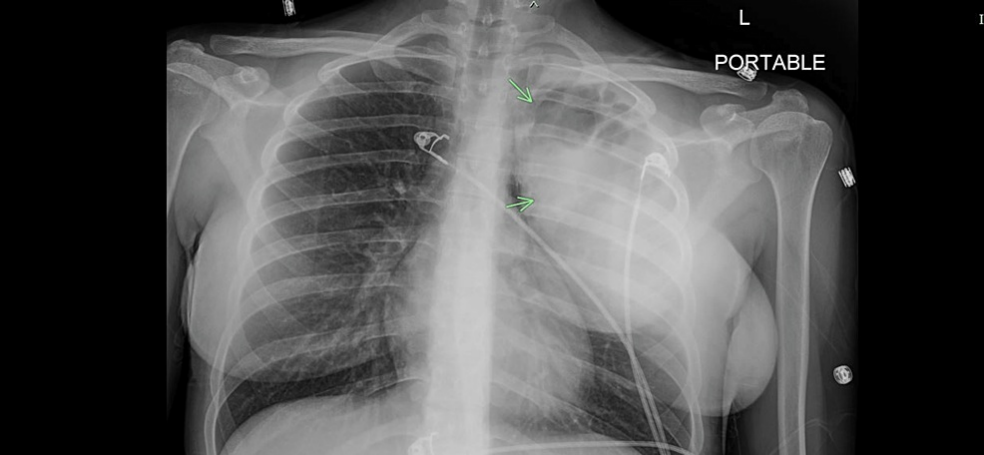
Chest X-ray on presentation showing the left-sided lung abscess

**Figure 2:**
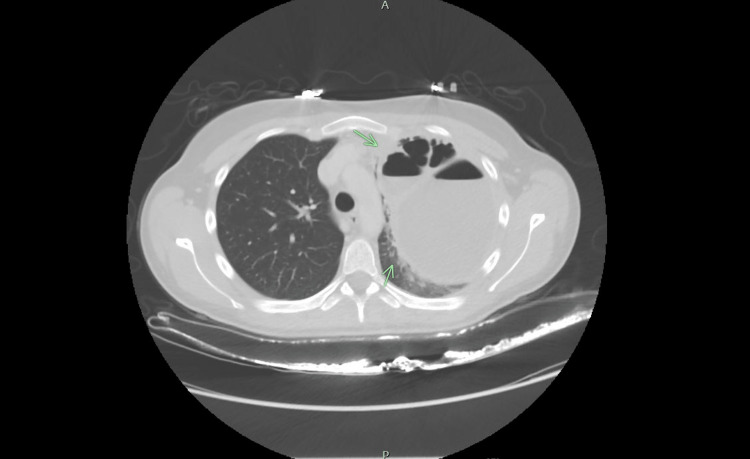
Chest CT on presentation showing a loculated left-sided lung abscess with air fluid levels

A 21-year-old female patient with a past medical history significant for anxiety presented to the emergency room with complaints of fever, cough and left-sided chest pain. She denied weight loss, night sweats, joint pain or swelling, rashes or lymphadenopathy. Home medications included escitalopram that she took regularly. Relevant recent history included symptoms of upper respiratory infection (URI) about two and a half weeks prior to presentation, including sore throat, nasal congestion and myalgias that improved in a week with conservative management. She denied smoking tobacco, consumption of alcohol or recreational substance use. She endorsed a history of electronic cigarette use with nicotine vapes, for six months prior to presentation. She had no known medication allergies, no recent travel history or sick contacts. Vitals were significant for mild sinus tachycardia on presentation with no other abnormalities. Physical examination was significant for coarse breath sounds with rhonchi in the left upper chest area. No evidence of poor dental hygiene was noted. Chest X-ray showed cavitary mass-like opacity in the left upper lobe (Figure 1). Subsequent computed tomography (CT) of the chest with intravenous contrast showed a complex, irregular, loculated fluid and gas cavity in the left upper lobe measuring 14.5 x 8.5 x 13.3 cm, along with adjacent patchy airspace consolidation (Figure 2). No evidence of other abnormalities was noted in the right lung or the pleural space.

**Figure 3:**
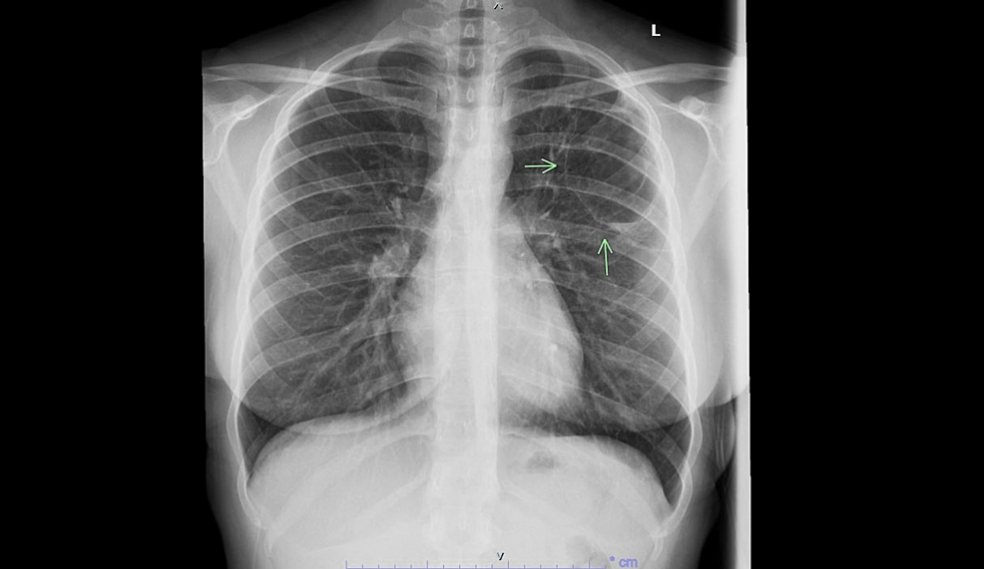
Chest X-ray done three weeks after antibiotics showing resolution of air fluid levels and improvement in the left lung abscess

**Figure 4:**
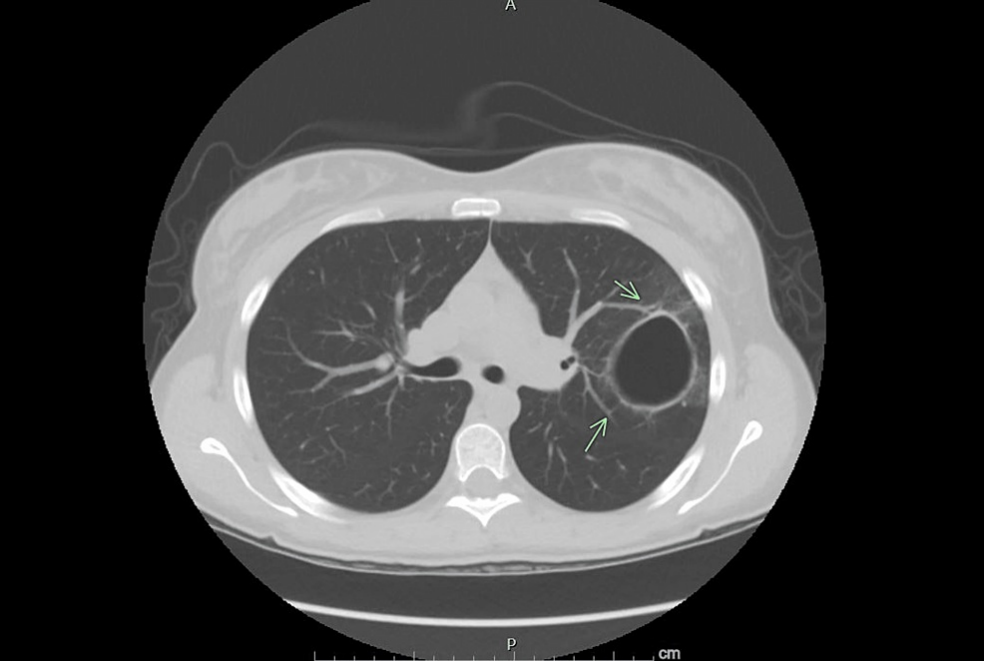
Chest CT done after five weeks of antibiotics showing significant improvement and resolution of air fluid levels

Laboratory data was significant for leukocytosis and elevated inflammatory markers including sedimentation rate and C-reactive protein. Urine drug screen and analysis were negative. COVID and influenza polymerase chain reaction testing was negative. Blood and sputum cultures were negative as well. Extended respiratory viral panel, Legionella and Streptococcal urinary antigen, beta-D-glucan, serum galactomannan, human immunodeficiency virus fourth-generation testing, and QuantiFERON tuberculosis test were negative. As the patient lived in an area endemic for Blastomyces and Histoplasma, respective serum antigen and serologies were tested that were negative. Based on the chest CT findings, a diagnosis of lung abscess was made. She was admitted to the inpatient medicine service and remained hemodynamically stable throughout her hospital course. She was seen by a multidisciplinary team including pulmonary, cardiothoracic surgery and infectious disease specialists. The patient was started on broad-spectrum intravenous antibiotics, vancomycin and piperacillin tazobactam and was monitored through daily complete blood counts, basic metabolic panel and liver function tests that were within normal limits. The QT interval was monitored with electrocardiogram and telemetry that stayed in the normal range. Her cough and chest pain improved within a few days of antibiotics, and she remained afebrile with resolving leukocytosis. Given the clinical improvement, the patient was discharged on linezolid and moxifloxacin to be taken for at least four to six weeks. The respective antibiotics were chosen to provided adequate Methicillin-resistant Staphylococcus aureus (MRSA) and anaerobic coverage. Second-line therapies including bronchoscopy and percutaneous abscess drainage were not considered due to the favorable clinical response and the fear of potential bronchopleural fistula formation. She was strongly advocated cessation of electronic cigarette use. The patient was followed up subsequently in the pulmonary and infectious disease clinic as an outpatient. She was noted to be doing well consistently and her antibiotics were further narrowed to amoxicillin clavulanate that she took for a total of six weeks. Repeat chest X-ray and chest CT showed a decreasing size of the cavitary mass in the left upper lobe, resolution of fluid in the cavity as well as resolution of patchy consolidative opacities (Figures 3, 4).

## Discussion

Lung abscess is a walled necrotizing infection involving the lung parenchyma, characterized by a cavitary lesion filled with fluid [1-2]. Based on the presence of predisposing lung abnormalities, lung abscesses are characterized as primary if the lung abscess develops in a normal lung, which accounts for 60% of the cases [2]. Secondary lung abscesses are seen in the setting of bronchial obstruction due to tumor, vascular compromise in the setting of emboli or impaired host defenses. The most common mechanism leading to primary lung abscess is aspiration of oropharyngeal contents [1]. This highlights the role of alcohol use and poor dental hygiene in the potentiation of lung abscess. The usual sequence of events includes aspiration pneumonitis that then progresses to liquefactive necrosis of tissues leading to fluid-filled cavity formation [2]. Lung abscesses have also been noted to develop from hematogenous dissemination in the setting of bacteremia or endocarditis. This usually results in multiple and bilateral disease, although unilateral/isolated lung abscess are also seen. Bronchial obstruction due to tumor, foreign body, and airway stenosis can result in post-obstructive pneumonia complicated by lung abscess. In rare cases, protracted and virulent cases of empyema can result in direct extension leading to lung abscess [2].

Known risk factors include poor dental/periodontal hygiene, alcoholism, recreational substance abuse, recurrent aspiration, diabetes mellitus, immunosuppressive therapy and recent Staphylococcal or Streptococcal pneumonia. Up to 50% of the patients do not have a positive microbiologic culture. Most commonly implicated bacteria include the oro-gingival anaerobes like *Peptostreptococcus*, *Prevotella*, *Bacteroides*, *Fusobacterium* and *Streptococcus anginosus*. Other bacteria like *Staphylococcus aureus*, *Klebsiella*, *Hemophilus*, *Streptococcus* and *Pseudomonas* are also responsible. Other rare causes include *Legionella*, *Nocardia *and *Actinomyces*. Nonbacterial pathogens including *Aspergillus* spp., *Histoplasma capsulatum*, *Blastomyces dermatitidis*, *Coccidioides* spp., *Mycobacterium tuberculosis *and atypical mycobacteria are also rarely implicated in relevant clinical situations [2-3].

Diagnosis is based on clinical symptoms as well as radiologic findings. Chest CT is the diagnostic test of choice as it not only clinches the diagnosis, but also helps to define the extent of the disease and identifies potential secondary causes responsible. First-line management includes expeditious initiation of antibiotics. About 80%-90% of the patients respond to antibiotics without the need for invasive procedures [4]. Antibiotics are usually administered for at least four to six weeks. Lack of improvement as indicated by persistent fevers, worsening respiratory symptoms in association with radiologic progression needs further consideration of procedures like bronchoscopy, endoscopic drainage via bronchoscopy, percutaneous transthoracic drainage, or surgical resection. The second-line approach for non-resolving lung abscesses is percutaneous aspiration and catheter drainage. Drainage of lung abscesses dates back to 1938, when it was used to drain tuberculous cavitary abscesses. However, with the advent of potent antibiotics, lung abscesses respond to medical therapy in 80%-90% of cases. A review of the literature showed acceptable outcomes for percutaneous drainage of lung abscesses not responding to medical treatment, with some studies reporting up to 80%-100% success rate [4-6]. This is especially useful in patients who have a significant morbidity and mortality risk for surgery. Some studies also recommend taking the size of the abscess into account when considering percutaneous drainage due to the risk of aspiration of abscess contents. In lung abscesses measuring 6-8 cm, not responding to initial antibiotic therapy, percutaneous drainage is recommended. The complication rate for percutaneous abscess drainage has been reported to be around 8% to 16%, with a mortality rate of around 4% [4-6]. Complications include catheter blockage necessitating repeat procedures, pneumothorax, hemothorax, hemoptysis and need for surgical intervention. One of the most feared complications is infection of the pleural space and resultant bronchopleural fistula. There have been reports with as high as 8% incidence of bronchopleural fistula cases of lung abscesses treated with percutaneous drainage [4-6]. In recalcitrant cases, surgical resection is considered, although it is associated with a mortality risk of up to 20%. Bronchoscopy has a potential role in cases of non-resolving lung abscesses to identify pathologies like tumors, foreign bodies, and airway stenosis. With the advent of various interventional pulmonary procedures, therapeutic options such as resection of endobronchial tumors, removal of foreign bodies and airway stents could be pursued that could eventually aid in clinical improvement.

In our patient, we suspect that she might have had a URI like influenza given her symptoms two to three weeks prior, which could have resulted in a secondary bacterial pneumonia leading to the lung abscess. There is a possibility that her use of electronic cigarettes with nicotine vapes could have contributed as well. Lung disorders caused by electronic cigarette use have been grouped under the term EVALI, e-cigarette or vaping product use-associated lung injury. It was initially recognized in 2019. As of February 2020, CDC has reported a total of 2807 hospitalized cases of EVALI [7]. Pulmonary manifestations of EVALI include acute respiratory distress syndrome, acute eosinophilic pneumonia, hypersensitivity pneumonitis, organizing pneumonia, lipoid pneumonia, pneumothorax and pneumomediastinum [8]. When reviewing the literature, we were able to find a few case reports of lung abscesses associated with electronic cigarette use [9-10]. Choi and Leungsuwan reported of a 21-year-old male with a two-week vaping history (one Juulpod every two weeks; Juul Labs, Inc., Washington DC) presenting with a lung abscess, which was successfully managed with antibiotics alone [9]. Chib et al. reported about a 50-year-old White male with a one-year vaping history of cannabinoids and nicotine, with a large lung abscess that required thoracotomy with drainage along with antibiotic treatment [10]. Electronic cigarettes contain various toxic chemicals and ingredients including vegetable glycerin, propylene glycol and vitamin E acetate (synthetic form of vitamin E). Inhalation of these vaporized agents could result in significant pulmonary inflammation due to their highly viscous nature [8]. Other postulated mechanisms include alteration of the pulmonary surfactant surface tension, dysregulated expression of cytokines and decreased immune function [8].

The pathophysiology of the development of lung abscess with electronic cigarette use remains unclear; however, we suspect that electronic cigarette use could contribute to significant parenchymal inflammation resulting in necrotizing pneumonia with lung abscess being the eventual result. Our case highlights the importance of a meticulous review of a patient's social history to prevent recurrences. Our patient had no predisposing conditions other than what is mentioned above and had no evidence of obvious immunosuppressed status. She had a rather large lung abscess that responded well to empiric antibiotic therapy with clinical and radiologic improvement.

## Conclusions

This case highlights the importance of a detailed history review, timely diagnosis and treatment of lung abscesses with appropriate antibiotics. The patient’s history of electronic cigarette use could have potentially contributed to the development of the lung abscess. Even large lung abscesses, as seen in our patient, respond to conservative management without the need for invasive procedures as they do carry significant risks. Nevertheless, it is of utmost importance to follow up with the patient to ensure continued clinical and radiologic improvement and prompt initiation of second-line measures in case of an inadequate therapeutic response.
